# Comparison of AirAngel® vs. Storz® videolaryngoscope and Macintosh® laryngoscope for endotracheal intubation training: prospective randomized crossover study

**DOI:** 10.1186/s12909-024-05388-0

**Published:** 2024-08-27

**Authors:** Şeyhmus Merter, Kamil Kayayurt, Dilek Kitapçıoğlu, Serpil Yaylaci

**Affiliations:** 1 School of Medicine, Emergency Department, Acibadem Mehmet Ali Aydinlar University, Istanbul, 34457 Turkey; 2 Center of Advanced Simulation and Education, Acibadem Mehmet Ali Aydinlar University, Istanbul, 34457 Turkey

**Keywords:** 3D printing, AirAngel, Intubation, Medical student, Video laryngoscope

## Abstract

**Background:**

For both normal and difficult airway management, VL is thought to be more effective. However, VL seems far from being offered as a standard option in both healthcare delivery and educational activities in low-income countries, considering its high costs. Therefore, three-dimensional(3D)printed VLs may be considered an alternative to conventional VLs in low-income countries and other places with limited resources. Our objective was to compare the efficacy of AirAngel 3D-printed VL (3D-PVL) with those of commercially available Storz® VL (SVL) and conventional Macintosh® laryngoscope (MCL) in normal and difficult airway scenarios in the hands of inexperienced users.

**Methods:**

This is a prospective randomized crossover manikin study that included 126 senior medical students with no experience in intubation. The effectiveness of all three laryngoscopy devices in the hands of inexperienced users was evaluated in terms of intubation time, glottic visualization, ease of use, endotracheal tube placement, and intubation success rate. Between 2020 and 2022, 126 last year medical students participated in the study.

**Results:**

MCL resulted in significantly longer intubation times than 3D-PVL and SVL in the difficult airway scenario, with no significant difference between 3DPVL and SVL (Wilcoxon test, *p* < 0.016; Bonferroni correction MCL: 28.54 s; SVL: 26.68 s; 3DPVL: 26.64 s). Both SVL and 3D-PVL resulted in significantly better Cormack − Lehane grades in both normal and difficult airway scenarios, and thus provided better glottic viewing than MCL, with no significant difference between 3D-PVL and SVL (Wilcoxon test, *p* < 0.016; Bonferroni correction, MCL: 1.73; SVL: 1.29; 3DPVL: 1.25). The SVL was the easiest device to use for normal airway scenarios (1: very easy, 5: very difficult), while the MCL was the most difficult (MCL: 2.64; 3DPVL: 1.98; SVL: 1.49). Conversely, no significant difference was found between 3DPVL and other devices in terms of ease of use in difficult airway scenarios and in terms of accurate placement of the endotracheal tube and successful intubation attempts.

**Conclusion:**

3D-PVL is a good educational and possible clinical alternative to conventional VL, particularly in places with limited resources, due to its low cost.

## Background

Video laryngoscopy (VL) has certain advantages over other techniques, such as better glottic imaging, higher intubation success in individuals with difficult airways, less force required for intubation, less cervical spine movement, and better image capture overall [1, 2]. The first attempt with VL has a higher rate of successful intubation compared to direct laryngoscopy (DL), particularly in individuals with difficult airways. Some experts have recently suggested that the VL should be accepted and used as the standard technique for imaging in all emergency intubations, not just difficult intubations [3, 4] However, VL is often not accessible in low-income countries because of its high costs. In particular, 35% of emergency departments have VL available in the United Kingdom (UK) compared to 7.8% in Turkey [5, 6] Additionally, hospitals may not be able to reach the devices even if they can cover the cost in cases where the demand is excessive [7].

3D printing is the process of creating 3-dimensional objects through a digital file, either by creating a new file or using an existing one. This rapidly developing technology is already used in many areas of daily life and has also been widely used in medical applications. 3D printers produce many expensive medical materials and devices at lower costs, enable personalized modeling (implants and prostheses), cell cultures, and surgical planning, and can also be used as educational material [8, 9]. One of these applications is 3D-printed VL (3D-PVL), which has become prominent in pandemic conditions. A 3D-PVL can be obtained for only 6 − 30 United States dollars (USD) compared to a VL that costs thousands of US dollars [4, 10, 11].

Moreover, a study comparing 3D-PVL with standard VL in difficult airway management for experienced practitioners demonstrated comparable success rates for both devices [4]. Furthermore, Although it has been suggested that the performance of custom-made video laryngoscopes is acceptable compared to conventional laryngoscopes, the evidence is inconclusive and few studies have been conducted [12]. Additionally, achieving intubation skills with the VL can be accomplished with fewer attempts compared to the MCL [13]. However, the literature presented no study that evaluated the efficiency of 3D-PVLs in inexperienced practitioners. Herein, our study aimed to investigate the effectiveness of 3D-PVLs in acquiring endotracheal intubation (ETI) skills in senior medical school students who are inexperienced users and compare 3D-PVL with standard MCL and VL.

## Methods

### Participants

This study is a prospective randomized crossover study. The study participants consisted of senior students enrolled in Acıbadem Mehmet Ali Aydınlar University Faculty of Medicine. The study was conducted at the Center of Advanced Simulation and Education (CASE) laboratory. Senior medical school students (SMSS) were invited via e-mail by the medical education coordination office of the university from November 2020 to January 2022. The SMSS were invited four times in total, twice in each academic year. Informed consent and the study plan were shared within the invitation e-mail. The e-mail stated that this study would not be graded, it would not have any effect on school success, participation is not compulsory, and they can participate in the study by making an appointment with CASE at any time during the academic year.

The sample size was calculated using G*Power Software® (Erdfelder, Foul, & Buchner, 1996) version 3.1.9.2. The statistical comparison was conducted according to the referenced publication [4]. The type-1 error and power of the test were chosen as 5% and 95%, respectively. Consequently, the targeted sample size was calculated as 126 considering randomization. The applications received after a sufficient number of students applied to the study were rejected, and no more students were recruited.

SMSS are routinely taking the standardized airway training module that includes only theoretical class lessons at the beginning of their sixth-grade medical school education. ETI training is given with both standard MCL and VL in this module, but there is no hands-on training on any mannequin or patients. A 30-min training video was prepared that indicated the differences between DL and VL, introduced difficult airway manikin (DAM)s and normal airway manikin (NAMs), and demonstrated ETI practices. All participants were required to watch this training video before the intervention.

### Randomization

Each ETI attempt could enhance the participant’s experience in the next attempt. Hence, the order of use of ETI devices was randomized to avoid experience-based bias. Six subgroups were needed to determine the order in which the SMSS would use the devices based on a permutation of three because three ETI devices were planned to be used in total. Accordingly, participants were randomized into six groups using the random team generator. Each group included 21 participants. Interventions were planned to be performed in the order specified below:

1. Group: MCL, 3D-PVL, SVL
2. Group: MCL, SVL, 3D-PVL
3. Group: SVL, 3D-PVL, MCL
4. Group: SVL, MCL, 3D-PVL
5. Group: 3D-PVL, MCL, SVL
6. Group: 3D-PVL, SVL, MCL

### Study protocol

Six experimental stations were set up. Each table was ensured to have one Trucorp Airsim® airway manikin. NAMs were used in the first three stations, and DAMs were used in the subsequent three. The interventions were to be conducted by the participants using NAMs first, followed by DAMs in the predetermined randomization order. Participants were assisted by an experienced assistant researcher in ETI during their attempts to extend the ETT, who also checked the intubation accuracy with a bag valve mask (BVM) at the end of the intervention.

Each participant was given 3 min before each intervention to be prepared for the device and manikin. It was aimed that the participants would not feel pressed for time and would not attempt to intubate quickly. To this end, 1 min was given to complete the intubation attempt. The intervention was started as the participant said, “I am ready,” and the assistant researcher vented the ETT with the BVM as the participant said, “I am done,” after he/she was sure that he/she had placed the ETT. The intubation time was assessed as the time elapsed between placing the laryngoscope in the mouth and the ventilation of the lungs or stomach. The intubation attempt was terminated although it was not completed yet and considered unsuccessful when the intubation time was over 1 min. Accurate ETT placement was evaluated by visual confirmation of the swelling of the lungs on the manikin. The intubation attempt was considered unsuccessful if the manikin’s stomach was filled with air. Conversely, the intubation attempt was considered successful if one or both lungs were filled with air.

While there is no precise definiton of an unsuccessful intubation attempt in the literature, the maximum recommended duration of an intubation attempt is considered as 30 s, and all conservative airway management maneuvers should have a limited period, because irreversible cerebral anoxia occurs within minutes. Hence, in our study; intubation attempts that lasted longer than 30 s were considered unsuccessful, although the participants were given 1 min to complete the intubation [14]. Thus, only intubation attempts that lasted ≤ 30 s, in which the ETT was accurately placed in the trachea, and the lungs were ventilated were considered successful. The rate of successful intubation attempts was calculated based on the intubation attempts deemed successful according to the said criteria.

The participants were asked to evaluate the ease of use of the device with a 5-point (from very easy to very difficult) Likert-type scale and to what extent they could view the vocal cords during the intervention according to the Cormack − Lehane classification, which was provided to the SMSS with visual material in the form of a Likert-type scale, after each intubation attempt. Thus, intubation times, ease of use scores, Cormack − Lehane grades, rates of accurate placement of ETT, and rates of successful intubation attempts were measured at the end of each intubation attempt with DAM or NAM using any of the three laryngoscopy devices.

### Materials

**Fig. 1:**
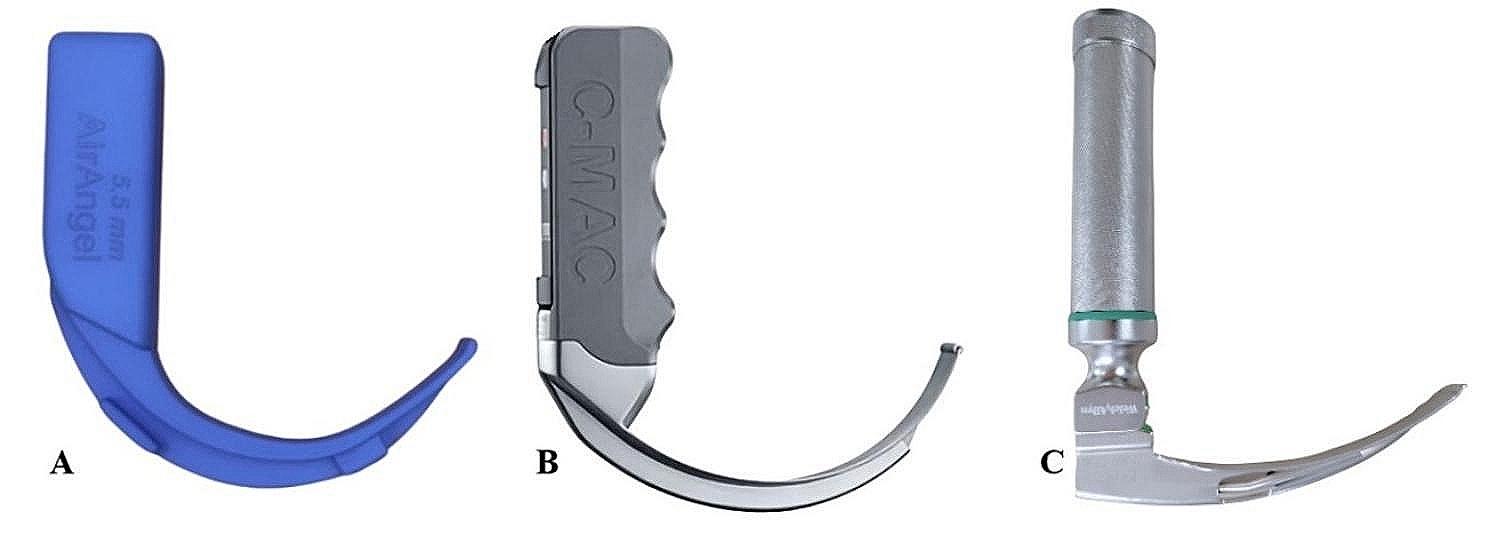
Laryngoscopes used in the study. **A**. 3D-printed video laryngoscope (Airangel®) **B**. Storz C-MAC video laryngoscope **C**. Macintosh laryngoscope (with blade no. 3). The blades of the video laryngoscopes are hyperangulated, as seen in A and B. The handle and blade of 3D-PVL are wider and thicker than other devices

The study utilized three different types of laryngoscopes, which are shown in Fig. 1. The globally accessible AirAngel® 5.5-mm standard adult laryngoscope model was chosen as the 3D-PVL blade model. AirAngel® is a 3D-PVL model produced with 3D printing technology, the digital file of which has been opened to public access by its designers, which has a hyperangulated blade and allows transferring the images to devices such as a phone, computer, or tablet with an integrated endoscope camera. The digital file containing the design codes of the model was downloaded from https://www.airangelblade.org/download. The AirAngel® 3D 5.5-mm standard adult laryngoscope model was printed with a Zortrax® M200 (Olsztyn, Poland) 3D printer at Acıbadem Mehmet Ali Aydınlar University Incubation Center at 80% density and as five layers from polylactic acid (PLA) material. The indentation at the end of the printed laryngoscope was suitable for integrating a 5.5-mm diameter camera (endoscope/borescope). The PLA material used in printing costs approximately 6 USD.

We mounted a 5.5-mm diameter, light-emitting diode (LED)-illuminated, International Protection Code (IP)67-certified, dustproof, and water-resistant borescope (Knmaster® ECO series endoscope camera) with a resolution of 640 × 480 pixels equipped with a 1/9inch complementary metal oxide semiconductor sensor and 1-m long universal serial bus (USB) connection to the printed laryngoscope for approximately 9 USD. The created 3D-PVL was connected to a smartphone compatible with USB On-The-Go, which is a feature that allows the phone to be used as a host allowing the use of other USB devices, with a 6.67-inch in-plane switching liquid crystal display with a resolution of 1080 × 2400 full high definition + pixels. A smartphone-supported 3D-PVL was obtained in this way.

A standard MCL and Storz® VL (SVL) consisting of 8401 AXC C-MAC® blade and 8403 ZX monitor was selected as the DL and VL devices to be compared with 3D-PVL, respectively. A size 3 Macintosh blade, which is the most commonly used in adults and compatible with 3D-PVL dimensions and design, was preferred for both MCL and SVL.

A Trucorp Airsim® (Trucorp, UK) task trainer was chosen as the airway manikin. The tongue of the manikin was fully inflated with 10 mL of air when the manikin was being used as a DAM, and the cervical spine screw was fully tightened to reduce head extension. No modifications were made when the manikin was used as a NAM. An endotracheal tube (ETT) with a standard 7.5-mm inner diameter was chosen. The stylet was placed in the ETT before the intervention. A new ETT and stylet were used in each intervention. Additionally, the ETTs were lubricated before each intervention.

### Statistical analysis

Participants’ demographic characteristics and their performance regarding the intervention according to the device used were recorded using Microsoft Excel® (Microsoft Corp., Redmond, WA, USA) software. Statistical analyses were performed using MedCalc® Statistical Software version 12.7.7 (MedCalc Software bvba, Ostend, Belgium). The probability (*p*) statistics of < 0.05 were deemed to indicate statistical significance. The mean and standard deviation (SD) values of the collected data were calculated. Descriptive statistics were used to express continuous variables (mean and SD, median, and minimum − maximum values). The Mann − Whitney U and Wilcoxon tests were used to examine the relationships between two independent and dependent nonnormally distributed continuous variables, respectively. Additionally, the Kruskal − Wallis and Friedman tests were used to analyze the relationships between more than two independent and dependent nonnormally distributed continuous variables, respectively. The chi-square or the likelihood ratio test, where appropriate, was used to examine the relationship between categorical variables. McNemar’s chi-square test was used to investigate the relationship between two dependent categorical variables.

## Results

A total of 126 participants were included, 77 (57.9%) participants were female, and the average age of the participants was 24.2 ± 1.17 years. All participants performed a total of six ETI attempts, two (one with a DAM and another with a NAM) with each of the three laryngoscopy devices. All participants completed the study steps and there were no participants excluded from the analysis. A total of 756 ETI attempts were made. The mean and SD values of the intubation times, Cormack − Lehane grades, ease of use scores, rates of accurate ETT placement, and successful intubation attempts were calculated (Table 1).

**Table 1:** The distribution of the variables investigated within the scope of the study by the ETI devices

Variables	ETI Devices			*p*-value*
	MCL	3D-PVL	SVL	
Normal Airway Scenario				
Intubation time ^a^	28.54 ± 9.1	26.64 ± 9.76	26.68 ± 8.83	**0.03**
Intubation time <30 s ^a^	24.29 ± 3.82	22.38 ± 3.52	23 ± 3.36	0.092
Cormack-Lehane grade (1–4) ^b^	1.73 ± 0.62	1.25 ± 0.45	1.29 ± 0.56	**< 0.001**
Ease of use (1–5) ^c^	2.64 ± 0.92	1.98 ± 0.85	1.49 ± 0.71	**< 0.001**
Rate of Accurate Placement of ETT	85.7% (108/126)	93.7% (118/126)	96.8% (122/126)	**0.005**
Successful Intubation Attempt Rate	82.5% (104/126)	83.3% (105/126)	86.5% (109/126)	0.627
Difficult Airway Scenario				
Intubation time ^a^	27.79 ± 8.2	28.02 ± 11.17	26.57 ± 10.22	**0.001**
Intubation time < 30 s ^a^	23.4 ± 3.19	22.46 ± 3.42	22.01 ± 3.41	**0.001**
Cormack-Lehane grade (1–4) ^b^	1.94 ± 0.73	1.33 ± 0.52	1.35 ± 0.67	**< 0.001**
Ease of use (1–5) ^c^	2.9 ± 1.05	2.25 ± 1.01	1.97 ± 1.03	**< 0.001**
Rate of Accurate Placement of ETT	77.8% (98/126)	88.1% (111/126)	89.7% (113/126)	**0.02**
Successful Intubation Attempt Rate	81.0% (102/126)	84.1% (106/126)	86.5% (109/126)	0.406

*Note* The values shown in the table are expressed as mean ± SD

*Abbreviations* ETI: endotracheal intubation, MCL: Macintosh laryngoscope, 3D-PVL: three dimensional-printed video laryngoscope, SVL: Storz video laryngoscope, ETT: endotracheal tube

The bold values indicate statistically significant values according to Friedman test, *p* < 0.05

* Friedman test, *p* < 0.05

^a^ The respective values are expressed in seconds and milliseconds

^b^ Likert-type scale: 1 = fully viewed glottis, 4 = no visible glottis structure

^c^ Numeric Rating Scale: 1 = very easy, 5 = very difficult

Comparison of the ETI devices in terms of intubation times of interventions completed in 1 min revealed significant differences between the ETI devices (Table 1). However, the post hoc pairwise subgroup analysis revealed a significant difference only between SVL and MCL and only with DAM, where the former yielded a significantly shorter intubation time (Table 2). Conversely, the comparison of the intubation times of interventions completed under 30 s revealed a significant difference in intubation times between the three ETI devices with DAMs but not with NAMs. The post hoc pairwise subgroup analysis performed to determine the pair that caused the significant difference indicated a significantly longer mean intubation time in MCL with DAMs compared to the other two devices but did not reveal any significant difference between 3D-PVL and SVL (Table 2).

**Table 2:** Comparison of ETI devices in terms of intubation time in normal and difficult airway scenarios

Variables	ETI Devices	Mean (± SD)	*p*-value*
Intubation time ^a^			
Normal Airway Scenario	MCL vs. SVL	(28.54 ± 9.1) vs. (26.68 ± 8.83)	0.025
	MCL vs. 3D-PVL	(28.54 ± 9.1) vs. (26.64 ± 9.76)	0.041
	SVL vs. 3D-PVL	(26.68 ± 8.83) vs. (26.64 ± 9.76)	0.808
Difficult Airway Scenario	**MCL vs. SVL**	(27.79 ± 8.2) vs. (26.57 ± 10.22)	**0.010**
	MCL vs. 3D-PVL	(27.79 ± 8.2) vs. (28.02 ± 11.17)	0.125
	SVL vs. 3D-PVL	(26.57 ± 10.22) vs. (28.02 ± 11.17)	0.756
Intubation time < 30 s ^a^			
Difficult Airway Scenario	**MCL vs. SVL**	(23.4 ± 3.19) vs. (22.01 ± 3.41)	**< 0.001**
	**MCL vs. 3D-PVL**	(23.4 ± 3.19) vs. (22.46 ± 3.42)	**0.004**
	SVL vs. 3D-PVL	(22.01 ± 3.41) vs. (22.46 ± 3.42)	0.548

*Abbreviations* ETI: endotracheal intubation, MCL: Macintosh laryngoscope, SVL: Storzvidelaryngoscope, 3D-PVL: three dimensional-printed video laryngoscope

The bold values indicate statistically significant values according to Wilcoxon test, *p* < 0.016 (Bonferroni correction)

*Wilcoxon test, *p* < 0.016 (Bonferroni correction)

^a^ The respective values are expressed in seconds and milliseconds

Comparison of the ETI devices in terms of ease of use scores and Cormack − Lehane grades with both DAMs and NAMs revealed significant differences between the ETI devices (Table 1). The post hoc pairwise subgroup analysis between the ETI devices in Cormack − Lehane grades indicated that both SVL and 3D-PVL resulted in significantly better Cormack − Lehane grades and hence provided better glottic viewing than MCL, but did not reveal any significant difference between 3D-PVL and SVL (Table 3). Additionally, the post hoc pairwise subgroup analysis between the ETI devices in ease of use scores revealed a significant difference between all ETI devices with the use of NAMs and between MCL and SVL with the use of DAMs. Accordingly, the easiest device to use with both NAMs and DAMs was the SVL, while the most difficult was the MCL (Table 3).

**Table 3:** Comparison of devices in terms of Cormack − Lehane grades and ease of use scores in normal and difficult airway scenarios

Variables	ETI Devices	Mean (± SD)	*p*-value*
Cormack − Lehane grade ^a^			
Normal Airway Scenario	**MCL vs. SVL**	(1.73 ± 0.62) vs. (1.29 ± 0.56)	**< 0.001**
	**MCL vs. 3D-PVL**	(1.73 ± 0.62) vs. (1.25 ± 0.45)	**< 0.001**
	SVL vs. 3D-PVL	(1.29 ± 0.56) vs. (1.25 ± 0.45)	0.492
Difficult Airway Scenario	**MCL vs. SVL**	(1.94 ± 0.73) vs. (1.35 ± 0.67)	**< 0.001**
	**MCL vs. 3D-PVL**	(1.94 ± 0.73) vs. (1.33 ± 0.52)	**< 0.001**
	SVL vs. 3D-PVL	(1.35 ± 0.67) vs. (1.33 ± 0.52)	0.976
Ease of use score ^b^			
Normal Airway Scenario	**MCL vs. SVL**	(2.64 ± 0.92) vs. (1.35 ± 0.67)	**< 0.001**
	**MCL vs. 3D-PVL**	(2.64 ± 0.92) vs. (1.33 ± 0.52)	**< 0.001**
	**SVL vs. 3D-PVL**	(1.35 ± 0.67) vs. (1.33 ± 0.52)	**< 0.001**
Difficult Airway Scenario	**MCL vs. SVL**	(2.9 ± 1.05) vs. (1.97 ± 1.03)	**< 0.001**
	MCL vs. 3D-PVL	(2.9 ± 1.05) vs. (2.25 ± 1.01)	0.040
	SVL vs. 3D-PVL	(1.97 ± 1.03) vs. (2.25 ± 1.01)	0.040

*Abbreviations* ETI: endotracheal intubation, MCL: Macintosh laryngoscope, SVL: Storzvidelaryngoscope, 3D-PVL: three dimensional-printed video laryngoscope

The bold values indicate statistically significant values according to Wilcoxon test, *p* < 0.016 (Bonferroni correction)

*Wilcoxon test, *p* < 0.016 (Bonferroni correction)

^a^ Likert-type scale: 1-fully viewed glottis, 4 = no visible glottis structure

^b^ Numeric Rating Scale: 1 = very easy, 5 = very difficult

A comparison of the ETI devices in terms of the rate of accurate placement of ETT revealed significant differences between the ETI devices (Table 1). The post hoc pairwise subgroup analysis between the ETI devices in the rate of accurate ETT placement indicated that SVL resulted in significantly higher rates of accurate ETT placement with NAMs than MCL (*p* = 0.004), but did not reveal any significant difference between the ETI devices with DAMs (McNemar’s test, *p* < 0.016 Bonferroni correction).

Last, a comparison of the ETI devices in terms of the rate of successful intubation attempts did not reveal any significant difference between the ETI devices, regardless of whether they were used with DAMs or NAMs (Table 1).

## Discussion

The study results revealed significantly better results with 3D-PVL and SVL in intubation time, glottic imaging, and ease of use in the normal airway scenario and glottic imaging in the difficult airway scenario than with MCL. Conversely, no significant difference was found between the three ETI devices investigated within the scope of this study in the rates of successful intubation attempts and accurate ETT placement variables, regardless of airway scenarios. Although video laryngoscopes provide superior glottic visualisation and ease of use, we do not consider these advantages to be clinically significant in reducing intubation time.

The duration of intubations performed by medical students with MCL reported in the literature ranges from 14 to 40.8 s for normal airway scenarios and 10.5 to 38 s for VL [15–19]. Although there was a statistically significant difference in intubation times between devices in our study, the fact that intubation times with all devices were shorter than the 30-second time frame recommended for successful intubation suggests that this difference is not clinically significant. In an analysis of related studies, Ray et al. found no significant difference between MCL and VL in a normal airway scenario [16]. Similarly, Pieters et al. reported that medical students achieved a shorter intubation time using a video laryngoscope in a normal airway scenario, but this was not statistically significant [15]. In contrast, Rendeki and Maharaj found that VL was superior to MCL in both scenarios and Shin et al. found that VL provided shorter intubation time after repeated attempts in the normal airway scenario but not in the difficult airway scenario. The differences between the studies may be attributed to differences in the methodologies of the respective studies and the use of different devices and manikins. In fact, one VL may even be superior to another VL in certain parameters [17, 18]. A meta-analysis of studies with real patients revealed shorter intubation times with the use of VL [20].

The results of the study showed that the SVL was the easiest ETI device to use of the three devices studied, while the MCL was the most difficult to use, regardless of the airway scenario. Similarly, relevant studies available in the literature reported that VL was easier to use than MCL in both normal and difficult airway scenarios [15–19]. The thicker blade and the less ergonomic blade handle created difficulties in using 3D-PVL in the difficult airway scenario based on the unstructured feedback received from the participants. These drawbacks were also mentioned by Gorman et al., and a channeled 3D-PVL with oxygen and suction features was developed to overcome these drawbacks [21].

SVL and 3D-PVL gave better results than MCL in terms of glottic imaging. A good glottic view may not be obtained in the DL technique when viewed from a limited mouth opening, even if the axes are aligned correctly. Conversely, the video camera helps the VL technique overcome obstacles such as the tongue, teeth, and limited mouth opening [22]. The camera at the end of the VL blade eliminates the need to align the oral, pharyngeal, and laryngeal axes. Previous studies have demonstrated that VL improved Cormack − Lehane grades and glottic imaging regardless of user experience [15–19, 23–26]. Although studies have shown that VL is superior in providing a better glottic view, it should be noted that these advantages do not always translate into successful intubation attempts [27].

Comparable results were obtained with all three ETI devices in terms of the rate of successful intubation attempts, regardless of airway scenarios. SVL and 3D-PVL provided significantly better glottic imaging than MCL, but this difference was not reflected in the rate of successful intubation attempts. Hence, obtaining a good glottic view does not always imply accurate ETT and successful ETI placement [28]. A good glottic view, as well as correct positioning and advancement of the ETT in the mouth and good hand-eye coordination, are required for accurate ETT and a successful ETI placement. The rate of successful intubation attempts in the manikin studies in which medical school students’ ETI successes were compared according to VL and MCL use were not significantly different, whereas a meta-analysis conducted by Nalubola et al. revealed that medical school students had higher intubation and first entry success in real patients, especially using channeled VL [15–18, 20]. Training medical school students first with VL while gaining ETI skills reportedly increased their intubation success by 14–19% and shortened the intubation time [29, 30].

An easily accessible option in the production of devices and tools is 3D printing technology, the costs of which are difficult to meet in many countries. Offering the designed devices open access as downloadable files would allow these devices to be produced globally when needed [11]. The need for 3D printing and VL production has increased because of the disadvantages of limited transportation during the pandemic period. However, studies on this subject remain limited. Studies available in the literature produced new VLs based on the existing VLs and DLs as examples, in addition to developing their own unique devices [4, 10, 11, 20, 31–34]. PLA material, which is considered biocompatible, was generally used in manufacturing these devices. Cohen compared 3D-PVL with MCL and reported the superiority of 3D-PVL over MCL in terms of first entry success rate, glottic imaging, and intubation time [33]. Papanaoum et al. revealed that 3D-PVL was more successful than MCL [31]. Similarly, Lambert et al. compared 3D-PVL with MCL and Pentax-AWS® and revealed that 3D-PVL is superior to MCL in terms of intubation time and success, glottic imaging, and ease of use [4]. Conversely, Ataman compared AirAngel® with Glidescope® and concluded that Glidescope was more successful in terms of the first entry success rate in both normal and difficult airway scenarios and intubation time in the normal airway scenario [10]. All the included participants in these studies were experienced practitioners. The heterogeneity in the outcomes reported by these studies might be because of the differences between the selected endpoints, the devices used (both standard VLs and 3D-PVLs), and manikins. A thorough literature review conducted during the writing of this study revealed no other study on the efficiency of 3D-PVL in inexperienced users.

It is crucial for medical students to learn simpler life-saving techniques like BVM rather than ETI. However, in countries such as Turkey where there is a shortage of general practitioners, newly graduated physicians are often required to work in emergency departments in rural areas without proper training in emergency patient management. This leaves them with little recourse but to apply advanced cardiac life support techniques without adequate training and minimal access to experienced physicians. This is especially concerning in countries with high physician shortages and underdeveloped regions. Therefore, training in ETI is also important to address this “Practitioner Gap” in emergency medicine [35]. Otherwise, many difficulties and obstacles exist in teaching ETI to medical school students. First, more than fifty attempts are needed to gain competency in conventional DL [13]. Then, performing this training on actual patients is often not possible, considering the busy operating room and emergency room conditions [29]. Gaining this competence to medical school students with VL offers safer, faster, and easier alternative training that can be completed in six or seven attempts [16, 36, 37]. The steps of the intervention can be controlled under the mentorship of an experienced laryngoscopist, and anatomical structures can be understood more easily due to the real-time feedback provided by the video camera [37]. However, the 40% access rate to VLs even in developed countries and much lower in developing and underdeveloped countries constitutes the biggest obstacle to giving this training [5, 6].

This study did not include a direct measurement that assessed the use of 3D-PVL in real patients. Studies on the subject matter reported that 3D-PVL should theoretically withstand a force of 100 N during ETI [32]. Other studies on PLA durability demonstrated that PLA could withstand a force of approximately 200 N [38]. Mendes et al. reported that the 3D-PVL model they developed for pediatric and adult patients could withstand forces of > 400 N[ [11]]. Gorman et al. reported that the 3D-PVL they produced complied with ISO7376:20 standards both in terms of rigidity and strength [21]. In comparison, > 750 manikin intubations were performed with the single 3D-PVL specifically produced for this study, yet no breakage or macroscopic deformation was observed. Additionally, 3D-PVL can be reused after sterilization by 1% Rely + OnTM Virkon solution or hydrogen peroxide or via methods such as gas plasma and gamma radiation [4, 21]. However, the number of sterilizations that the structural properties of 3D-PVL will withstand is not reported. In sum, 3D-PVL was not validated on real patients, but the findings suggest its use in real patients in the near future because of the fast pace of technological developments in this field.

### Limitations of the study

This study is a manikin study. Therefore, directly adapting the study results to real patients is impossible. It would be unethical to recommend using this device, which results from a new idea and the durability and efficiency of which have yet to be standardized on real patients other than a manikin. The structure of the manikins used in the study can simulate the airway anatomy of a patient with a normal airway to a certain extent. However, a manikin can simulate only one of the difficult airway scenarios, i.e., cervical immobilization, because of the numerous difficult airway scenarios in real life. The devices and manikins used in this study were adult models and, thus, unsuitable for assessing pediatric airways.

The video laryngoscopes utilized in our study were of the hyperangulated type, as clearly illustrated in Fig. 1. Notwithstanding, it is important to note that the difficult airway scenario created by inflating the mannequin’s tongue could potentially skew the results, leading to an overestimation of the superior effectiveness of 3D-PVL and SVL over MCL. It is crucial to keep in mind that there exist a wide range of difficult airway situations that cannot be replicated on a manikin.

This study was not a blind study as the type of laryngoscope used could not be concealed from the participant or the researcher measuring the intubation times There are two possible outcomes that can arise from a situation like this. Firstly, if the individual fails at their initial attempt or is stressed out due to the time constraints, it can lead to underperformance. Secondly, there is the Hawthorne effect which is characterized by higher than normal productivity levels when trainees are being followed. Although robust endpoints (tracheal and oesophageal intubations and intubation times) were clearly defined in the trial, the Hawtorne effect, which refers to the possibility that subject monitoring improves outcomes, may have contributed to the trial results being better than they would have been otherwise [39].

Additionally, the study participants, who were inexperienced, might have gained experience with each intubation attempt, which might have improved the results obtained at subsequent stations, as pointed out in the literature [19]. This situation was foreseen during the study’s planning phase, and the device order was randomized to negate the said effect. However, it is unclear how much the results were affected.

The Cormack − Lehane classification was used to evaluate glottic imaging. This classification was developed for DL and thus the subjective nature of the evaluations made with this classification might have affected the results [26]. Although the CL classification has been preferred in many similar studies in the literature [38, 39], other classifications that may give different results from the classical CL classification, such as the modified CL classification, intubation difficulty score (IDS), percentage of glottic opening (POGO), have not been evaluated [40–42].

It is recommended to use a malleable stylet for all emergency intubations [14]. In our study, participants used a stylet for ETI attempts in both airway scenarios. However, it should be noted that although the use of a stylet facilitates the intubation process, it may lead to complications such as pharyngeal, tonsillar and alveolar bleeding, tracheal and bronchial rupture and haemopneumothorax [43].

The principle of creating material in layers is the basis of 3D printing, and the layers’ thickness can affect the material’s strength. Additionally, 3D-printed video laryngoscope models do not come out of a standard production line and thus the 3D printer used in production may affect the material quality which is a limitation of this technology.

The qualities of the images produced by 3D-PVL and SVL may have been different because these two techniques have different video camera resolutions. 3D-PVL has a resolution of only 640 × 480 pixels, whereas SVL has a resolution of 1280 × 800 pixels. However, the difference in terms of pixels did not cause a significant difference in glottic imaging on the manikin. The manufacturer declares that the endoscope camera is resistant to fogging and wetness, but the effects of secretions and liquids available in a real patient on the images produced thereof are unknown. However, the study results did not reveal any disadvantage for that matter, when performing ETI training on a manikin.

## Conclusions

A shorter intubation time was achieved in 3D-PVL than in MCL in the difficult airway scenario, and 3D-PVL offered comparable results to SVL. Conversely, 3D-PVL produced significantly better glottic images than MCL and comparable glottic images to SVL in both normal and difficult airway scenarios. No significant difference was found between the three techniques in ease of use in the difficult airway scenario. However, 3D-PVL was superior to MCL in ease of use in the normal airway scenario. No significant difference was found between the techniques in the rates of accurate ETT placement and intubation success, regardless of the airway scenario. For any variable investigated within the scope of the study, 3D-PVL was not inferior to MCL, and it produced comparable results to SVL. In conclusion, 3D-PVL can be a good educational tool and clinical alternative to conventional VLs, particularly in countries with limited resources, given its low cost.

## Data Availability

All data obtained and analysed in this study are presented in the article.

## Abbreviations

BVM: Bag valve mask
CASE: Center of Advanced Simulation and Education
DAM: Difficult airway manikin
DL: Direct laryngoscopy
ETI: Endotracheal intubation
NAM: Normal airway manikin
SD: Standard deviation
SMSS: Senior medical school students
UK: United Kingdom
USD: United States dollars
VL: Video laryngoscopy
